# Structure-based engineering of anti-GFP nanobody tandems as ultra-high-affinity reagents for purification

**DOI:** 10.1038/s41598-020-62606-7

**Published:** 2020-04-10

**Authors:** Ziyue Zhang, Yao Wang, Yu Ding, Motoyuki Hattori

**Affiliations:** State Key Laboratory of Genetic Engineering, Collaborative Innovation Center of Genetics and Development, Department of Physiology and Biophysics, School of Life Sciences, Fudan University, 2005 Songhu Road, Yangpu District, Shanghai, 200438 China

**Keywords:** Biological fluorescence, Ion channels, X-ray crystallography, Protein purification

## Abstract

Green fluorescent proteins (GFPs) are widely used in biological research. Although GFP can be visualized easily, its precise manipulation through binding partners is still burdensome because of the limited availability of high-affinity binding partners and related structural information. Here, we report the crystal structure of GFPuv in complex with the anti-GFP nanobody LaG16 at 1.67 Å resolution, revealing the details of the binding between GFPuv and LaG16. The LaG16 binding site was on the opposite side of the GFP β-barrel from the binding site of the GFP-enhancer, another anti-GFP nanobody, indicating that the GFP-enhancer and LaG16 can bind to GFP together. Thus, we further designed 3 linkers of different lengths to fuse LaG16 and GFP-enhancer together, and the GFP binding of the three constructs was further tested by ITC. The construct with the (GGGGS)_4_ linker had the highest affinity with a K_D_ of 0.5 nM. The GFP-enhancer-(GGGGS)_4_-LaG16 chimeric nanobody was further covalently linked to NHS-activated agarose and then used in the purification of a GFP-tagged membrane protein, GFP-tagged zebrafish P2X4, resulting in higher yield than purification with the GFP-enhancer nanobody alone. This work provides a proof of concept for the design of ultra-high-affinity binders of target proteins through dimerized nanobody chimaeras, and this strategy may also be applied to link interesting target protein nanobodies without overlapping binding surfaces.

## Introduction

Green fluorescent proteins (GFPs) are among the most extensively studied and widely used genetic tools in biological and medical research. Since jellyfish-derived GFP has the advantages of fast maturation to functional fluorescent proteins in almost all organisms, a lack of additional cofactors, easy observation by standard fluorescence microscopy, preserved function when fused with target proteins to obtain chimeric proteins, and utility as a genetic tool with GFP expression maintained in the offspring, it has been extensively investigated and widely used to visualize dynamic biological processes *in vivo* and *in vitro*^1–5^. Many GFP-tagged animal and plant models have also been established. Although the visualization of GFP and GFP fusion proteins is easy, their manipulation is still cumbersome.

One way to manipulate GFP is to find binding partners, including antibodies, nanobodies and designed ankyrin repeat proteins (DARPins)^6^. Since the antibody is relatively large, disulfide bond formation is needed during the *in vivo* assay, and the high propensity for aggregation hinders its application. DARPins can also recognize target proteins with similar specificities and affinities to those of antibodies; since they are extremely stable, they are widely used as intracellular sensors of protein conformations and as crystallization scaffolds^7^. Other small protein binders have also been developed, including monobodies^8^, affibodies^9^, anticalins^10^ and nanobodies.

Among these protein binders, nanobody technology is the most promising because it can be adapted for use in humans and ultimately utilized as a therapeutic reagent^11^. Nanobodies are relatively small in size, resistant to denaturants and organic solvents capable of tolerating harsh purification and biochemical assay conditions, and expressed in all cell types with high solubility^12–17^. In 1993, Hamers Casterman *et al*. reported a specific class of light chain-deleted antibodies in camels^18^. The heavy chain variable region (VHH) is the smallest antigen binding unit and can be further cloned as nanobodies. Typically, monomeric nanobodies are only 12–15 kD, while the conventional IgG antibody is approximately 150 kD. The height of a nanobody is approximately 4.8 nm, and the diameter is approximately 2.2 nm^12^. Since GFP is the most important genetic marker for biological research, several groups have generated different nanobodies targeting GFP or its variants^19–21^.

Although several nanobodies targeting GFP have been used to purify or control the fluorescence intensity, the strongest reported binding affinity is not subnanomolar and therefore is not yet strong enough to purify low-abundance GFP-tagged endogenous proteins. However, efforts in structural-based directed evolution or library-based display technology did not significantly improve the affinity of the GFP nanobody. Therefore, we sought to improve the affinity by linking different nanobodies that recognize different portions of GFP with small peptide linkers. However, the lack of structural information on the detailed binding sites hindered the design and application of manipulation of GFP or GFP fusion proteins by high-affinity antibodies. We first solved the crystal structure of the complex formed by GFPuv and the LaG16 nanobody, which showed that GFPuv’s LaG16 binding site was on the opposite side of GFP from the GFP-enhancer nanobody. We designed 3 linkers of different lengths to connect the GFP-enhancer and LaG16 nanobody and tested their binding affinity to GFP. The (GGGGS)_4_ linker was the best, and the chimaeric nanobody was further crosslinked to agarose gel to test its utility in protein purification. The purification of the membrane protein GFP-zfP2X4 showed that the chimeric nanobody performed better than the single nanobody.

## Results

### Structure determination of the LaG16-GFPuv complex

**Figure 1:**
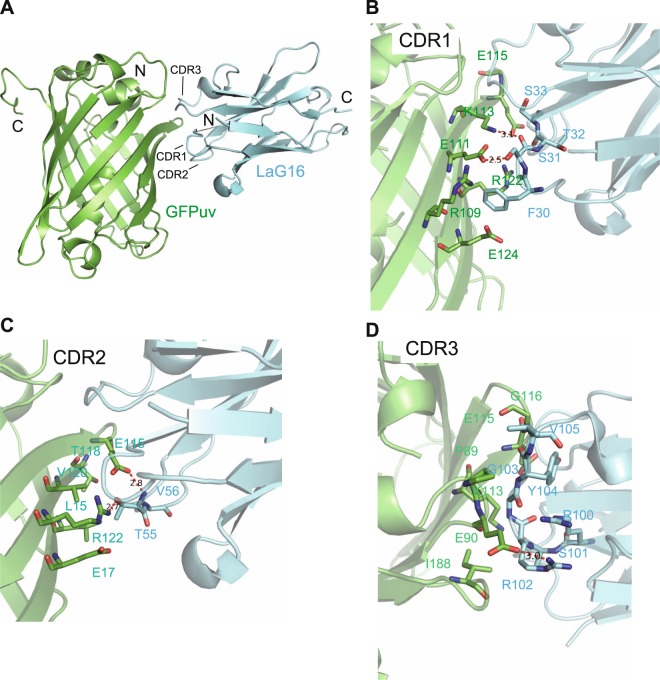
Structure of the LaG16-GFPuv complex. (**A**) The overall structure of the LaG16-GFPuv dimer. GFPuv is shown in green, and LaG16 is shown in blue. (**B**–**D**) View of LaG16’s CDR1-GFPuv, CDR2-GFPuv, and CDR3-GFPuv interaction interfaces. Amino acids participating in the interfaces are shown in stick representation. The red dash indicates the major hydrophilic interactions between LaG16’s CDR1, CDR2 and CDR3 and GFPuv.

We determined the crystal structure of the GFPuv-LaG16 complex at 1.67 Å resolution (Fig. 1), in which all three CDRs of LaG16 form specific contacts with GFPuv. The expanded view of the binding surface between the 3 CDR domains and GFPuv shows hydrophilic, hydrophobic, and electrostatic interactions, which determine the high specificity and affinity of LaG16 for GFPuv. CDR1 (Fig. 1B) has hydrogen bonds between Ser31 of LaG16 and Glu111 and Lys113 of GFPuv. In CDR2 (Fig. 1C), Thr55 of LaG16 forms hydrogen bonds with Glu115 and Arg122 of GFPuv, which is crucial to the orientation of the binding interface. Val56 of LaG16 interacts with Leu15 of GFPuv through hydrophobic interactions, contributing to the high specificity of dimer formation. In CDR3 (Fig. 1D), Arg102 of LaG16 and Glu90 of GFPuv form a salt bridge, resulting in the high affinity of LaG16 to GFPuv. In addition, hydrophobic interactions are shown between Val105 of LaG16 and Pro89 of GFPuv. We also compared the structure of GFPuv in the GFPuv-LaG16 complex we determined (PDB ID: 6LR7) and the GFPuv monomer (PDB ID: 6IR7). The total RMS deviation was only 0.250 Å, and thus, the binding of nanobody LaG16 did not significantly change the overall structure of GFPuv.

### LaG16 and GFP-enhancer can bind to GFPuv at the same time

**Figure 2:**
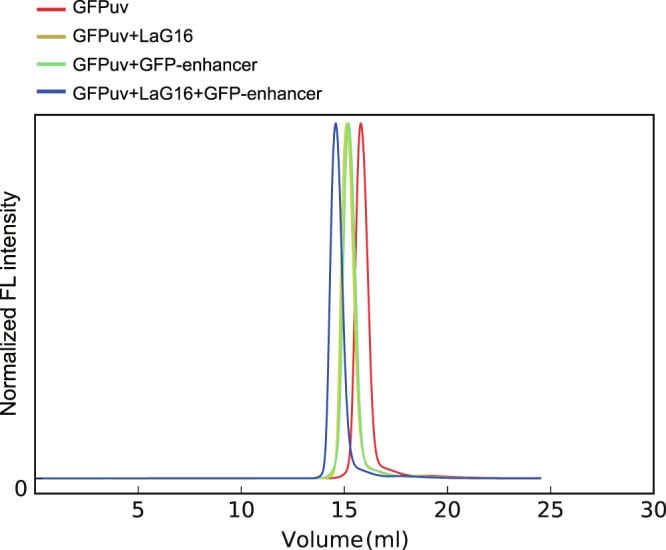
GFP-enhancer and LaG16 can bind to GFPuv simultaneously, as confirmed by the FSEC method. The fluorescence of GFPuv was detected, and the heights of the peaks were normalized. Mixtures of GFPuv+LaG16, GFPuv+GFP-enhancer, GFPuv+LaG16+GFP-enhancer, GFPuv and nanobodies were incubated at mass ratios of 1:1.5, 1:1.5, and 1:1.5:1.5, respectively.

To confirm that LaG16 and GFP-enhancer can bind to GFPuv noncompetitively *in vitro*, we used the FSEC method^22^. After the addition of only one kind of extra nanobody (LaG16 or GFP-enhancer) to GFPuv, the peak representing GFPuv emission exhibited an obvious shift compared to the peak of GFPuv alone, proving that either LaG16 or GFP-enhancer can bind to GFPuv (Fig. 2). In the sample with both LaG16 and GFP-enhancer added, all the GFPuv was incorporated into the LaG16-GFPuv-GFP-enhancer triple complex, whose peak shows a larger shift than that of the GFPuv-nanobody dimer. Therefore, the FSEC method confirmed that LaG16 and GFP-enhancer can bind to GFPuv at the same time.

### Design of fusion nanobody based on the triple structure model

**Figure 3:**
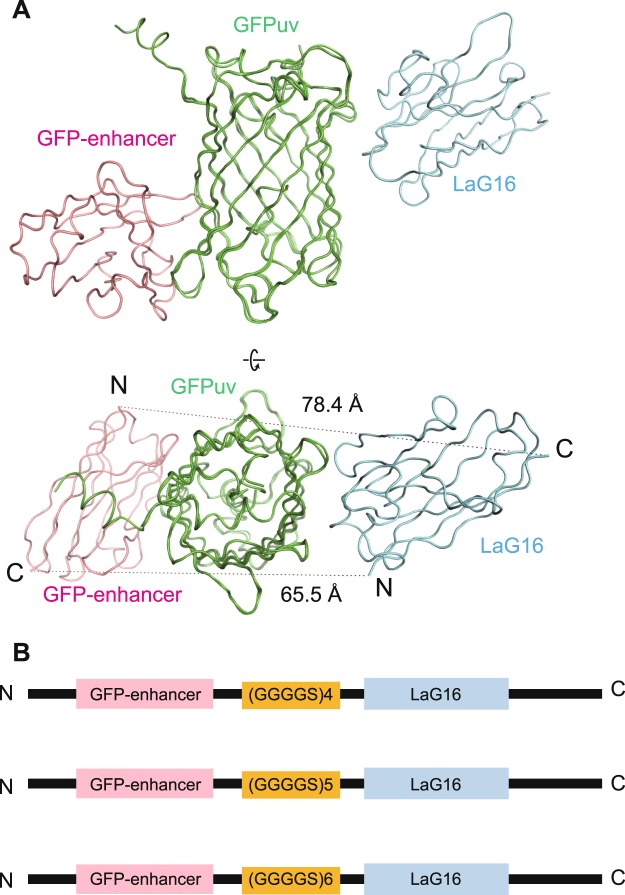
Design of fusion nanobodies. (**A**) Overall modelled structure of the GFP-enhancer-GFPuv-LaG16 complex based on crystal structures of GFP-enhancer-GFP (PDB ID: 3K1K) and GFPuv-LaG16 (PDB ID: 6LR7). GFP-enhancer, GFPuv and LaG16 are coloured pink, green and blue, respectively. The distance from the C terminus of the GFP-enhancer to the N terminus of LaG16 and the distance from the N terminus of the GFP-enhancer to the C terminus of LaG16 were measured by PYMOL software. (**B**) Design of the vector carrying the two nanobodies and a linker. Different numbers of (GGGGS) repeats were inserted to connect the C terminus of the GFP-enhancer and the N terminus of LaG16, forming a fusion nanobody.

As nanobodies are powerful tools for the purification of GFP-tagged proteins and there are many commercialized nanobody resin products used for purification, we attempted to produce a fusion nanobody with heightened affinity to GFPuv for use in purifying protein with improved yield. Repeated (GGGGS) amino sequences can form a flexible linker between two proteins, and one turn of (GGGGS) has been found to be 19 Å long^23^. Based on the modelled structure of the LaG16-GFPuv-GFP-enhancer triple complex (Fig. 3A), we calculated that the distance from the N terminus of LaG16 to the C terminus of the GFP-enhancer (65.5 Å) is shorter than the distance from the N terminus of the GFP-enhancer to the C terminus of LaG16 (78.4 Å). Thus, we decided to add several (GGGGS) repeats between the N terminus of LaG16 and the C terminus of the GFP-enhancer. Too short a linker will cause tension when the fusion nanobody binds to GFPuv, while too long a linker will decrease the stability of the fusion nanobody. We added 4/5/6 (GGGGS) repeats between the two nanobodies (Fig. 3B) and used the ITC method to select the best fusion nanobody with the most suitable linker.

### Determination of the affinity constant between GFPuv and anti-GFP nanobody tandems

**Figure 4:**
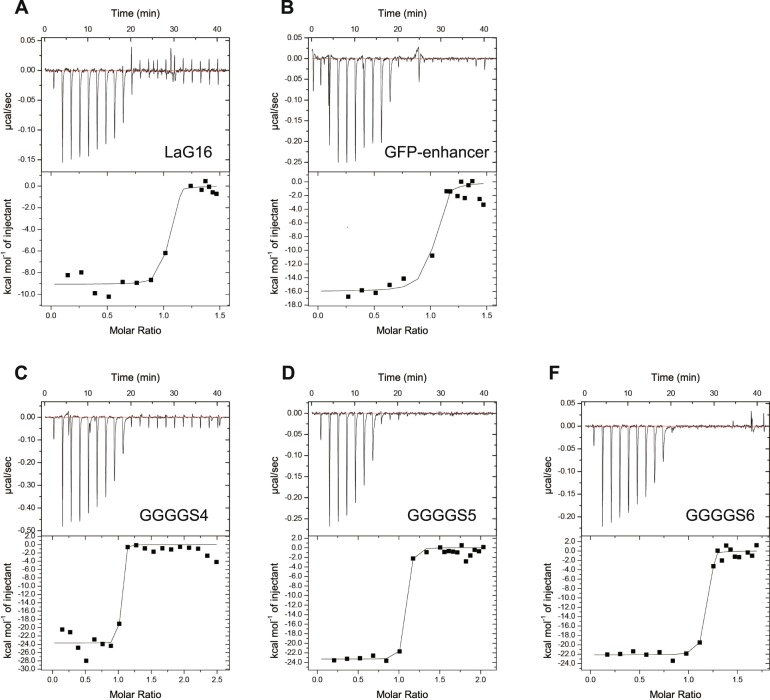
Analysis of the affinity of different nanobodies to GFPuv through ITC. The raw ITC data and fitted curves are shown. The data were obtained by injecting (**A**) LaG16, (**B**) GFP-enhancer, (**C**) GGGGS_4_ (GFP-enhancer-(GGGGS)_4_-LaG16), (**D**) GGGGS_5_ (GFP-enhancer-(GGGGS)_5_-LaG16), or (**E**) GGGGS_6_ (GFP-enhancer-(GGGGS)_6_-LaG16) into GFPuv in the cell.

To examine whether the fusion nanobodies had higher affinity for GFP than the individual ones, we measured the binding affinity of LaG16, GFP-enhancer, GGGGS_4_, GGGGS_5_ and GGGGS_6_ to GFPuv (GGGGS_4_, GGGGS_5_, and GGGGS_6_ are the abbreviations of the fusion nanobodies GFP-enhancer-(GGGGS)_4_-LaG16, GFP-enhancer-(GGGGS)_5_-LaG16, and GFP-enhancer-(GGGGS)_6_-LaG16, respectively) (Fig. 4, Table 1). GFPuv exhibits a Kd of 6.7 nM with LaG16 and a Kd of 24.3 nM with GFP-enhancer. All fusion nanobodies showed a greater affinity to GFPuv than the single GFP-enhancer or LaG16 nanobody. The Kd values of GGGGS_4_, GGGGS_5_ and GGGGS_6_ to GFPuv were 0.5 nM, 0.6 nM, and 1.2 nM, respectively. When the linker was too long, the LaG16 and GFP-enhancer in the tandem nanobodies could be treated as two separate and unrelated molecules and would not affect each other. When the linker length was properly optimized, as one of the nanobodies bound to GFP antigen, the linker restricted the movement of the tandem-linked nanobody to rotation and twisting in a small range. When the second nanobody’s GFP binding site was nearby, there was a greater chance to simultaneously bind two nanobodies to one GFP molecule. As the fusion nanobody with the shortest linker, GGGGS_4_, showed the highest affinity with GFPuv, we chose GGGGS_4_ for the nanobody-coupled resin application.

**Table 1:** The binding specificity of different nanobodies to GFPuv.

	n	ΔΗ° (kcal/mol)	−TΔS° (kcal/mol)	ΔG° (kcal/mol)	K_D_ (nM)
GFP-enhancer	0.99 ± 0.02	−16.0 ± 0.7	5.5	−10.5 ± 0.7	24.3 ± 17.0
LaG16	0.99 ± 0.03	−9.1 ± 0.3	2.0	−7.1 ± 0.3	6.7 ± 14.5
GGGGS_4_	0.99 ± 0.02	−23.7 ± 0.8	10.9	−12.8 ± 0.8	0.5 ± 2.3
GGGGS_5_	1.01 ± 0.01	−23.3 ± 0.4	10.6	−12.7 ± 0.4	0.6 ± 0.6
GGGGS_6_	1.1 ± 0.01	−22.1 ± 0.4	9.8	−12.3 ± 0.4	1.2 ± 0.7

### Application of the GGGGS_4_ nanobody for membrane protein purification

**Figure 5:**
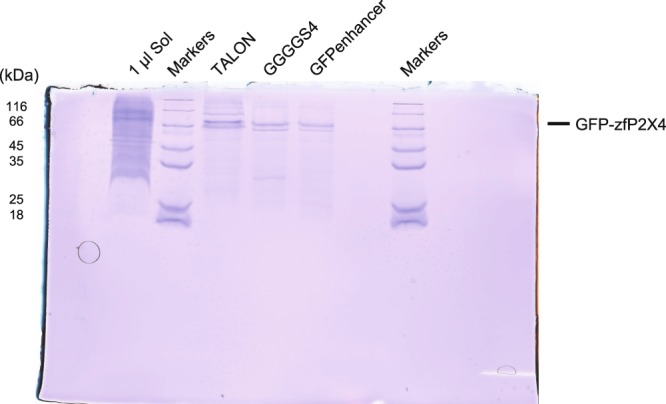
Analysis of zfP2X4 purification using nanobody-coupled resin or TALON resin through SDS-PAGE gel. The first sample line shows the solubilization solution of the cell membrane, containing all kinds of membrane proteins. The numbers on the left indicate the molecular weight of the marker. The 3rd, 4th and 5th lines are the protein purified using TALON, GGGGS_4_ (GFP-enhancer-(GGGGS)_4_-LaG16)-coupled and GFP-enhancer-coupled resins, respectively. The bands for GFP-zfP2X4 are indicated on the right.

We coupled GGGGS_4_ or GFP-enhancer to NHS-activated Sepharose4 Fast Flow resin and used the resin to purify GFP-tagged zebrafish P2X4 receptor^24^, a membrane protein, from pelleted SF9 cell membrane. The eluted protein was analysed by SDS-PAGE (Fig. 5). The solubilized cell membrane showed a very weak band of GFP-P2X4 in the gel, while the eluted solution showed a strong band of GFP-zfP2X4, which means that both GGGGS_4_-coupled resin and GFP-enhancer-coupled resin can catch the GFP-tagged protein with high specificity. However, the GGGGS_4_-coupled resin had a higher yield, as the intensity of GFP-zfP2X4 analysed by ImageJ software was at about 1.5x that obtained with the GFP-enhancer (Table 2). We also performed and compared the purifications by the anti-GFP resins and by the TALON his-tag purification resin, which was previously employed for P2X4 purification^24^. The results showed that the anti-GFP resins yielded a much higher purity than the TALON resin (Fig. 5, Table 2).

**Table 2:** Purification of GFP-tagged zfP2X4 from 7.67 ml of cultured cells.

	Total Protein (μg)	Purity (%)	Concentration (mg/ml)	Volume (μl)	Yield (μg)
TALON	32.5	36.3	0.650	50	11.8
GGGGS_4_	10.7	69.3	0.214	50	7.42
GFP-enhancer	6.75	75.2	0.135	50	5.08

## Discussion

In this work, we determined the structure of the GFPuv-LaG16 complex and revealed the interaction between the CDR regions of LaG16 and GFPuv. The model of the GFP-enhancer-GFPuv-LaG16 triple complex and FSEC testing confirmed that GFP-enhancer and LaG16 can bind to GFPuv at the same time. More importantly, we designed the fusion nanobody GGGGS_4_ (GFP-enhancer-(GGGGS)_4_-LaG16) and tested it for purification of a GFP-tagged protein, obtaining a higher yield than the original GFP-enhancer.

As GFP fusion expression screening techniques such as FSEC^24^. have been widely used in membrane protein structural biology, affinity purification using anti-GFP nanobodies has also become increasingly popular. In particular, after the Cryo-EM revolution, FSEC screening of functional membrane proteins suitable for single-particle Cryo-EM by fusion with GFP became the general strategy. However, the contents of important GFP-tagged membrane protein complexes in cultured mammalian cells are relatively low, and the yield of affinity purification by crosslinking a single GFP nanobody affinity resin with nanomole-scale affinity is not sufficient for Cryo-EM. Tandem nanobody binding to GFP with subnanomolar affinity significantly improved the yield and overcame this problem. The fusion nanobody GGGGS_4_ in our study may provide a better choice for the purification of GFP-tagged proteins, particularly those with very low expression.

Additionally, direct manipulation of the *in vivo* target protein level is gradually becoming popular because DNA- and RNA-level manipulation, including knockout, knockdown and gene editing, is indirect, and unwanted side effects may cause incorrect results. Since GFP has been widely used to generate cell lines and animal models, controlling the expression level of target proteins fused with GFP may also simplify *in vivo* manipulation. Successful attempts have included directed protein degradation through anti-GFP nanobodies fused to E3 ligase. Several groups^25,26^ have proven the usefulness of the nanobody-controlled degradation of specific nuclear proteins in mammalian cells and zebrafish embryos. With ultra-high-affinity nanobody chimaeras, the efficiency of this approach may be further improved.

## Methods

### Vector construction

The ORFs of LaG16, GFP-enhancer nanobodies and GFPuv were synthesized and inserted into the pET-28b vector between the NdeI and BamHI restriction sites by GENEWIZ, Inc. For the construction of fusion tandem nanobodies, (GGGGS)_4_, (GGGGS)_5_ and (GGGGS)_6_ were inserted between the C terminus of the GFP-enhancer and the N terminus of LaG16 by GENEWIZ, Inc. (Table 3).

**Table 3:** The DNA sequences encoding the linkers of the (GGGGS)_4_, (GGGGS)_5_, and (GGGGS)_6_ constructs.

Name	DNA Sequences encoding related linker
(GGGGS)_4_	GGCGGTGGCGGTAGCGGTGGTGGCGGCAGCGGCGGCGGTGGTAGCGGCGGTGGTGGCAGC
(GGGGS)_5_	GGCGGTGGCGGTAGCGGTGGTGGCGGCAGCGGCGGCGGTGGTAGCGGCGGTGGTGGCAGCGGTGGTGGTGGTAGT
(GGGGS)_6_	GGCGGTGGCGGTAGCGGTGGTGGCGGCAGCGGCGGCGGTGGTAGCGGCGGTGGTGGCAGCGGTGGTGGTGGTAGTGGTGGCGGCGGCAGC

### Expression and purification

The plasmid was transformed into *E. coli* Rosetta (DE3) cells and plated on Luria Bertani (LB) medium with 1.25% agar, 30 μg/ml kanamycin and 30 μg/ml chloramphenicol. Colonies of transformed Rosetta (DE3) cells were inoculated into LB medium. The next day, 1% of the cells cultured overnight were added to LB medium with 30 μg/ml kanamycin and incubated with shaking at 37 °C until the OD 600 nm reached approximately 0.6. Protein expression was induced by adding 0.5 mM isopropyl-b-D-1-thiogalactopyranoside (IPTG), and the cells were grown at 18 °C with shaking (220 rpm). Cells were harvested after 16 hours by centrifugation at 4000 × g for 10 min. Cell pellets were suspended in TBS (50 mM Tris pH 8.0, 150 mM NaCl) containing 1 mM phenylmethylsulfonyl fluoride (PMSF) and lysed using a High Pressure Homogenizer (JN-3000 PLUS, JNBIO, China) at 1,000 bar 5 times. The cell debris and inclusion bodies were removed by centrifugation at 35000 × g for 30 min. The supernatant was applied to a Ni-NTA (Qiagen) column pre-equilibrated with buffer A (50 mM Tris-HCl pH 8.0, 150 mM NaCl, 30 mM imidazole). The mixture was rotated at 4 °C for 1 hour, the beads were washed to remove unbound protein with 10 CV of buffer A, and the protein was eluted with elution buffer (50 mM Tris-HCl pH 8.0, 150 mM NaCl, 300 mM imidazole). The eluted protein’s His8 tag was removed in a 3.5 kD dialysis membrane (spectra/Por 7) by HRV3C protease at a mass ratio of target protein:HRV3C = 10:2 overnight at 4 °C. Then, 500 ml of dialysis buffer was added to remove imidazole (50 mM Tris-HCl pH 8.0, 150 mM NaCl, 15 mM imidazole). The dialysis buffer was exchanged again during dialysis. On the next day, the digested protein was applied to a column equilibrated with dialysis buffer. Then, the column was rotated at 4 °C for 1 hour, and the flow-through fraction was collected and concentrated to 10 mg/ml using an Amicon Ultra 10 K filter (Millipore). Next, the protein was applied to a Superdex 75 Increase size-exclusion column (GE Healthcare) equilibrated with SEC buffer (20 mM HEPES pH 7.0, 150 mM NaCl). The target recombinant proteins with the tag removed were collected and concentrated to 10 mg/ml.

### Crystallization

LaG16 nanobodies and GFPuv (GFPuv: LaG16 = 1: 1.2; GFPuv: LaG16: GFP-enhancer= 1: 1.2: 1.2) were mixed and rotated at 4 °C for 1 hour. Then, the mixture was centrifuged at 41600 × g for 20 min, and the supernatant was applied to a Superdex 75 Increase size-exclusion column (GE Healthcare) equilibrated with SEC buffer (20 mM HEPES pH 7.0, 150 mM NaCl). The fractions containing the dimer/triple complex were collected and concentrated to 10 mg/ml. The crystals were obtained by vapour diffusion over a solution containing 0.3 M NaCl, 0.01M Tris-HCl 8.0, 27.5% w/v PEG4000 (for GFPuv-LaG16 complex).

### Data collection and structure determination

All data sets were collected at SPring-8 BL32-XU (Hyogo, Japan). The data sets were processed with XDS programs^27^. The structure of the GFPuv-LaG16 complex was determined by molecular replacement using the Phaser program from the CCP4 crystallography package^28,29^ with PDB ID code 6IR6 for GFPuv and the LaG16 model built based on chain C of 3K1K as the search models. The refinement was performed by Refmac^30^ and Phenix^31^, and the model was further adjusted by COOT^32^. The related figures were drawn using PyMOL^33^. The structure refinement statistics are summarized in Table 4.

**Table 4:** Data collection, phasing and refinement statistics.

Data collection	
Wavelength (Å)	1.000
Space group	*P*2_1_
Cell dimensions	
*a*, *b*, *c* (Å)	48.4, 41.8, 81.7
*α*, *β*, *γ* (°)	90, 91.9, 90
Resolution (Å)*	42.22–1.67 (1.73–1.67)
*R*_sym_*	0.127 (0.609)
*I*/σI*	11.6 (2.04)
Completeness (%)*	99.34 (94.21)
Redundancy*	7.0 (3.9)
CC_1/2_ (%)*	99.4 (70.8)
**Refinement**	
Resolution (Å)	1.67
No. reflections	37934
*R*_work_/*R*_free_	20.5/23.8
No. atoms	
Protein	2883
Ligands	22
Solvent	170
B-factors	
Protein	13.2
Ligands	12.6
Solvent	27.0
R.m.s. deviations	
Bond lengths (Å)	0.024
Bond angles (°)	1.8
Ramachandran plot	
Favoured (%)	99.15
Allowed (%)	0.85
Outliers (%)	0

*The highest resolution shell is shown in parentheses.

### Isothermal titration calorimetry

The binding of nanobodies to GFPuv was measured using a Microcal ITC2000 microcolorimeter (GE Healthcare) at 20 °C. GFPuv and related nanobodies were purified as described above. We injected 280 μl of 5 μM GFPuv into the cell, and the ligand solution was 75 µM nanobody. The ligand was injected 20 times (0.4 μl for injection 1, 2 μl for injections 2–20), with 120 s intervals between injections. The baseline was obtained by adding ligand to SEC buffer. Before analysis, the baseline determined from GFPuv-nanobody samples was subtracted. The data were analysed by the Origin7 software package (MicroCal). Measurements were repeated two times, and similar results were obtained.

### Coupling nanobodies to NHS-activated sepharose4 fast flow beads

Since the activated NHS resin will form a covalent band with Tris buffer, we used HEPES instead of Tris during purification. Nanobodies were expressed as described above. Cells were harvested by centrifugation at 4000 × g for 10 min. Cell pellets were suspended in HBS (20 mM HEPES pH 7.0, 150 mM NaCl) containing 1 mM PMSF and lysed using a High Pressure Homogenizer (JN-3000 PLUS, JNBIO, China) at 1,000 bar 5 times. The cell debris was removed by centrifugation at 35000 × g for 30 min. The supernatant was applied to a Ni-NTA (Qiagen) column pre-equilibrated with buffer A (20 mM HEPES pH 7.0, 150 mM NaCl, 30 mM imidazole), and the mixture was rotated at 4 °C for 1 hour. Then, the beads were washed with 10 CV of buffer A, and the protein was eluted with elution buffer (20 mM HEPES pH 7.0, 150 mM NaCl, 300 mM imidazole). The eluate was placed in a dialysis membrane (spectra/Por 7) to remove extra imidazole using dialysis buffer (20 mM HEPES pH 7.0, 150 mM NaCl). Then, the digested protein was concentrated to 10 mg/ml.

The NHS-activated Sepharose4 Fast Flow beads (GE Healthcare) were washed with 20 CV of cold 1 mM HCl and equilibrated with 10 CV of HBS (20 mM HEPES pH 7.0, 150 mM NaCl). The resin was incubated with nanobodies at a ratio of nanobody:resin =1 mg:100 μl at 4 °C overnight. The next day, the resin was washed with 10 CV of blocking buffer (0.1 M Tris-HCl pH 8.0) and then incubated for 2 hours at 25 °C to quench unreacted NHS sites. Then, the resin was washed with six cycles of wash buffers (buffer 1: 0.1 M Tris-HCl pH 8.0, 0.5 M NaCl, and buffer 2: 0.1 M sodium acetate, 0.5 M NaCl, pH 4.0). The anti-GFP resin was equilibrated in storage buffer (10 mM Tris-HCl pH 8.0, 150 mM NaCl) and stored at 4 °C. The binding capacity of the anti-GFP nanobody coupled with Sepharose was tested and found to be approximately 0.5 mg of recombinant GFP per millilitre.

### Anti-GFP and TALON resins were used to purify GFP-tagged zfP2X4

The expression and cell disruption of zfP2X4 were performed as described previously^24^. One hundred and eighty microlitres of pelleted membrane (presumably containing approximately 60 μg of GFP-tagged zfP2X4) was solubilized with 180 µl of S buffer (50 mM Tris-HCl pH 8.0, 150 mM NaCl, 30% glycerol, 4% DDM, 1 mM PMSF, 5.2 μg/ml aprotinin, 2 μg/ml pepstatin A, 2 μg/ml leupeptin, and 0.5 U/m apyrase. Then, the unsolubilized membrane was removed by ultracentrifugation at 41600 × g for 20 min at 4 °C.

The supernatant was divided evenly into three 1.5 ml EP tubes and incubated with 50 μl of anti-GFP resin (GFP-enhancer or GGGGS_4_ tagged resin) equilibrated with wash buffer Ι (50 mM Tris-HCl pH 8.0, 150 mM NaCl, 15% glycerol, 0.05% DDM) or 50 μl of TALON resin (Takara) equilibrated with wash buffer ΙΙ (50 mM Tris-HCl pH 8.0, 150 mM NaCl, 15% glycerol, 0.05% DDM, 25 mM imidazole). The mixture was rotated at 4 °C for 1 hour, and then the resin was centrifuged at 200 × g for 2 min to remove the unbound protein. Then, 100 μl of wash buffer was added to the resin and centrifuged at 200 × g for 2 min to remove the supernatant. This washing step was repeated 5 times. Finally, the resin was applied to a spin column (Micro Bio-Spin Columns, BIO-RAD), and GFP-tagged zfP2X4 was eluted by 50 μl of elution buffer Ι (0.1 M glycine pH 2.0, for anti-GFP resin) or elution buffer ΙΙ (50 mM Tris-HCl pH 8.0, 150 mM NaCl, 15% glycerol, 0.05% DDM, 250 mM imidazole, for TALON resin). Before collecting the elution from anti-GFP resin, 5 μl of 1 M Tris-HCl pH 8.0 buffer was added to the collecting tube to adjust the pH.

### Quantitative analysis of the bands in the SDS-PAGE gel

The intensities of the bands in the SDS-PAGE gel were quantified using ImageJ software. The band of the target protein was selected manually, and the intensity of the peak was obtained using the “magic wand” tool in ImageJ.

### Verification of complex formation by the FSEC method

Two micrograms of purified GFPuv in SEC buffer was incubated with 3 μg of nanobody at 4 °C by rotating for 30 min and then centrifuged at 41600 × g for 20 min. The supernatant was loaded onto a Superdex 200 Increase size-exclusion column (GE Healthcare) equilibrated with SEC buffer (20 mM HEPES pH 7.0, 150 mM NaCl). The eluent was passed through a fluorometer (excitation, 480 nm; emission, 510 nm for GFP fluorescence). The data were processed and normalized with FSECplotter software.

### Ethical approval and informed consent

No human or vertebrate samples were used.

## Data Availability

All data generated and analyzed during the current study are available from the corresponding author. The atomic coordinates and structure factors have been deposited in the Protein Data Bank (http://www.pdb.org) with the accession code 6LR7. The plasmid construct of the fusion nanobody GGGGS_4_ has been deposited to AddGene (http://www.addgene.org/) (Addgene ID: 140442).
